# Value analysis of serum β -HCG, AMH, and P/E_2_ for predicting freeze-thaw embryo transfer outcomes in patients with thin endometrium

**DOI:** 10.12669/pjms.41.4.9980

**Published:** 2025-04

**Authors:** Yani Yan, Jian Zhang, Weiwei Li, Xiurong Yin

**Affiliations:** 1 Yani Yan Department of Reproductive Medicine, Maternity & Child Care Center of Qinhuangdao, Qinghuangdao 066000, Hebei, China; 2 Jian Zhang Department of Psychiatry, Jiulongshan Hospital of Qinghuangdao, Qinghuangdao 066000, Hebei, China; 3 Weiwei Li Department of Reproductive Medicine, Maternity & Child Care Center of Qinhuangdao, Qinghuangdao 066000, Hebei, China; 4 Xiurong Yin Department of Reproductive Medicine, Maternity & Child Care Center of Qinhuangdao, Qinghuangdao 066000, Hebei, China

**Keywords:** β-HCG, AMH, Frozen-thawed embryo transplantation, Pregnancy, P/E_2_, Thin endometrium

## Abstract

**Objective:**

To analyze the value of serum human chorionic gonadotropin (β-HCG), anti-Mullerian hormone (AMH), progesterone (P)/ estradiol (E_2_) in predicting the pregnancy outcome of frozen-thawed embryo transfer (FET) cycle in patients with low profile endometrium.

**Methods:**

In this retrospective study, 120 patients with low-profile endometrium who received FET therapy in the reproductive center of Maternity & Child Care Center of Qinhuangdao from October 2019 to February 2023 were included. Serum β-HCG, AMH, and P/E_2_ levels were measured on the day of FET transplantation. After 30 days they were divided into pregnancy group and non-pregnancy group according to whether they were pregnant. The risk factors affecting the cycle pregnancy rate of FET were analyzed. The values of β-HCG, AMH, P/E_2_ and the combination of the three in predicting the periodic pregnancy rate of FET in patients were analyzed.

**Results:**

Logistic regression analysis showed that age (≥35 years), type of infertility (primary infertility), transplanted fetus, moderate and severe intrauterine adhesions and P, P/E_2_ levels were risk factors for the periodic pregnancy rate of FET (OR > 1, P<0.05). The ROC curve showed that the AUC of serum β-HCG, AMH, P/E_2_ and their combination to predict the pregnancy rate of FET cycle in patients with thin endometrium was 0.83(95%CI:0.741-0.910), 0.86(95%CI:0.788-0.922), 0.75(95%CI:0.662-0.838), and 0.88(95%CI:0.807-0.947).

**Conclusion:**

Serum β-HCG, AMH, and P/E_2_ are closely related to the cycle pregnancy rate of FET in patients with thin endometrium. Therefore, dynamic monitoring of the changes of three indicators should be conducted clinically.

## INTRODUCTION

Freeze-thaw embryo transfer (FET) is a modified assisted reproductive technology (ART) based on traditional in vitro fertilization-embryo transfer (IVF-EF). It can be used as a supplementary suggestion for cancelling fresh embryo cycle transfer under adverse conditions such as uterine effusion and ovarian hyperstimulation. It has the advantages of high embryo utilization rate, short cycle and few complications, and can maximize the pregnancy rate involved.^1^,^2^ The embryo-endometrial dialogue theory is considered to be the classic theory of successful pregnancy, which states that a successful pregnancy requires good endometrial tolerance, a good quality embryo and synchronization.^3^

Endometrial thickness is the main index to assess endometrial tolerance, which not only reflects the endometrial status, but also the transformation of endometrium from the proliferative phase to the secretory phase is the key to successful embryo implantation.^4^ At present, there is no uniform clinical conclusion on the definition of thin endometrium, and it is mostly considered that endometrial thickness less than 7 or 8 mm on the day of HCG injection or ovulation is diagnostic.^5^ Relevant data show that the incidence of thin endometrium in couples treated with ART ranges from 1.5% to 9.1%, which is an important reason affecting the reproductive prognosis of ART.^6^ Therefore, timely detection of biological markers affecting the reproductive prognosis of ART in patients with thin endometrium is important in early screening of those at high risk for poor prognosis and improving pregnancy outcomes. AMH is a glycoprotein dimer, which induces mullerian duct degeneration, inhibits male mullerian duct development and regulates germ cell and gonadal development. Compared with luteinizing hormone (LH) and follicle-stimulating hormone (FSH), AMH is more useful in directly reflecting the reserve capacity of primordial follicles, and it is less regulated by the hypothalamic-pituitary-gonadal axis and fluctuates less with the menstrual cycle.^7^,^8^ In this study, one hundred and twenty patients with thin endometrium treated with FET in the our Fertility Centre of Maternity & Child Care Center of Qinhuangdao were selected to analyse the value of serum β-HCG, AMH and P/E_2_ in predicting pregnancy outcome of FET cycles in patients with thin endometrium.

## METHODS

This was a retrospective study. A total of 120 patients with low-profile endometrium who received FET therapy in the reproductive center of Maternity & Child Care Center of Qinhuangdao from October 2019 to February 2023 were included. Serum β-HCG, AMH, and P/E_2_ levels were measured on the day of FET transplantation. The patients were examined by transvaginal ultrasonography 30–35 days after embryo transplantation. They were divided into pregnancy group(n=77) and a non-pregnancy(n=43) group according to whether pregnant. The researcher selected 120 patients with thin endometrium, aged 24-42 years, mean (32.18±3.25) years; infertility age 2-8 years, mean (4.86±2.15) years; type of infertility: primary infertility 10, secondary infertility 110; number of previous pregnancies 0-4, mean (1.90±1.12); body mass index (BMI) 17-25 kg/m^2^, mean (21.69±1.24) kg/m^2^.

### Ethical Approval:

The study was approved by the Institutional Ethics Committee of Maternity & Child Care Center of Qinhuangdao (No.: QFYLL2018010; date: May 10, 2018).

### Inclusion criteria:

- Participants had an endometrial thickness of less than 8mm on the day of FET endometrial transformation.
- Met the FET indications in the Expert Consensus on Laboratory Operations for Human In Vitro Fertilization-Embryo Transfer.^9^
- Had not received progestogen therapy in the one month prior to enrollment.
- Patients between the ages of 20 to 45.
- Had regular menstrual cycles with dominant follicles.
- Voluntarily signed the informed consent form.

### Exclusion criteria:

- Participants with abnormal uterine development.
- Combined endometriosis, fibroids, adenomyosis or uterine polyps.
- Endocrine abnormalities.
- Previous history of adverse pregnancy.
- Suspected pregnancy.
- Unexplained vaginal bleeding; abnormal coagulation.

Participants were to take estradiol valerate (Huazhong Pharmaceutical Co., Ltd., Specification: 1 mg, State Drug Administration H42021397), 3 mg in the morning and 3 mg in the evening, continuously from day three of the menstrual cycle. On day 12-14 of the menstrual cycle, determine the thickness of the endometrium under ultrasound and adjust the dosing according to the thickness of the endometrium. If the thickness was less than or equal to 7 mm, add estradiol tablets/estradiol digestrol tablets (manufacturer: Abbott Biologicals B.V., specification: 1 mg/tablet, quantification number: H20110159), one red tablet in the morning and one in the evening for vaginal administration. The maximum administration time of estradiol valerate was 20 days. If the thickness of the endometrium was less than 8 mm, you might request to abandon the cycle. When the endometrium was greater than or equal to 8 mm and E_2_ greater than or equal to 200 pg/ml, progesterone injections were given intramuscularly to transform the endometrium (until day six for blastocyst transfer and until day four for oocyte transfer) at a dose of 60 mg/d. Embryos were implanted on day four or six of endometrial transformation and post-transfer estrogen therapy was given with luteal supplementation until the day of pregnancy test.

### Serum laboratory indicators:

Five ml of venous blood was collected from the patient on the day of FET transplantation on an early morning fast (12 hours fast), and the serum was separated by centrifugation for 10 minutes (centrifugation rate of 3000 r/min, radius of 8 cm), and the supernatant was taken and placed in a refrigerator at -80°C for measurement. Serum β-HCG, AMH, P and E_2_ levels were measured by electrochemiluminescence and the P/E_2_ values were calculated using a Roche Cobas e601 electrochemistry immunoluminescence instrument.

### Statistical analysis:

Epidata software was used for data entry and SPSS 23.0 software was used for data processing. T-test was used for measurement data conforming to normal distribution, and χ^2^ test was used for counting data expressed as percentages; logistic model analysis was used for multi-factor, and ROC curve was used to analyse the predictive value, α=0.05, and when P<0.05, the difference was statistically significant.

## RESULTS

There were 77 successful pregnancies (64.17%) and 43 non-pregnancies (35.83%) in 120 FET cycles in patients with thin endometrium. There were no statistically significant differences in FET cycles, number of embryos transferred, BMI, number of previous pregnancies, and endometrial thickness in the pregnancy group compared to the non-pregnancy group (P<0.05), while there were statistically significant differences in age, years of infertility, type of infertility, whether a blastocyst was transferred, and whether a combination of moderate to severe uterine adhesions was distributed in the pregnancy group compared to the non-pregnancy group (P<0.05). The results are shown in Table-I. β-HCG, AMH and E_2_ were all higher in the pregnant group than in the non-pregnant group, while P and P/E_2_ were lower than in the non-pregnant group (P < 0.05). The results are shown in Table-II.

**Table-I:** Comparison of basic information of the two groups (n, *χ̅*±*S*).

Factors		Pregnancy group(n=77)	Non-pregnancy group(n=43)	P
Age	24-35 years	63	28	0.043
	≥ 35 years	14	15
Years of infertility	< 2 years	39	24	0.038
	2-6 years	32	10
	> 6 years	6	9
Type of infertility	Primary infertility	3	7	0.022
	Secondary infertility	74	36
FET cycle	< 2	56	32	0.841
	≥ 2	21	11
Number of embryos transferred	< 2	35	20	0.911
	≥ 2	42	23
Whether to transplant a blastocyst	Blastocyst	53	19	0.008
	Non-blastocyst	24	24
Whether to combined moderate to severe uterine adhesions	Exist	8	12	0.016
	Null	69	31
BMI(kg/m^2^)		22.05±1.35	21.38±1.22	0.061
Numbers of previous pregnancies		1.90±1.18	1.89±1.08	0.964
Thickness of inner membrane (mm)		7.28±0.55	7.19±0.52	0.383

**Table-II:** Comparison of laboratory indicators between the two groups (*χ̅*±*S*).

Factors	Pregnancy group(n=77)	Non-pregnancy group(n=43)	P
β-HCG(U/L)	6.26±1.47	4.52±1.49	0.000
AMH(ng/ml)	2.75±0.40	2.13±0.38	0.000
P(ng/ml)	40.05±5.00	42.81±4.97	0.004
E_2_(pg/ml)	159.26±10.16	149.35±11.42	0.000
P/E_2_	0.25±0.04	0.29±0.04	0.000

The indicators that differed between Table-I and Table-II (age, years of infertility, type of infertility, whether a cystic fetus was transplanted, whether a combination of moderate to severe uterine adhesions, AMH, P, E_2_, P/E_2_) were used as independent variables and assigned values, and the pregnancy status of 120 FET cycles was used as the dependent variable (1=non-pregnant, 0=pregnant), and the independent variables were screened by logistic regression analysis with the forward introduction method. The variable testing method was the likelihood ratio test. The results showed that age (≥35 years), type of infertility (primary infertility), transplanted cystic fetus, and moderate to severe uterine adhesions and P and P/E_2_ levels were risk factors (OR >1, P < 0.05) and β-HCG, AMH and E2 levels were protective factors (OR<1, P< 0.05) for the pregnancy rate in FET cycles. The results are shown in Table-III and IV.

**Table-III:** Multi-factor logistic analysis of the assignment of each variable.

Independent variables	Variables’ Description	Assignment	
		Zero point	One point
Age	Categorical variable	≥ 12 years	< 12 years
Years of infertility	Categorical variable	Null	Exist
Type of infertility	Categorical variable	Have Stationary work	None Stationary work
Whether to transplant a blastocyst	Categorical variable	Null	Exist
Whether to combined moderate to severe uterine adhesions	Categorical variable	Null	Exist
β-HCG	Continuous variable	-	-
AMH	Continuous variable	-	-
P	Continuous variable	-	-
E_2_	Continuous variable	-	-
P/E_2_	Continuous variable	-	-

**Table-IV:** Multi-factorial logistic analysis of pregnancy rates in 120 FET cycles.

Independent variables	β	Standard error	Wald	P	OR	95% CI
Age	0.880	0.436	4.082	0.043	2.411	1.027-5.661
Years of infertility	0.678	0.446	2.313	0.128	1.969	0.822-4.716
Type of infertility	1.568	0.719	4.750	0.029	4.796	1.171-19.644
Whether to transplant a blastocyst	1.026	3.393	6.797	0.009	2.789	1.290-6.032
Whether to combined moderate to severe uterine adhesions	1.206	0.505	5.698	0.017	3.339	1.241-8.984
β-HCG	-0.822	0.174	22.293	0.000	0.439	0.312-0.618
AMH	-4.070	0.775	27.588	0.000	0.017	0.004-0.078
P	0.109	0.040	7.622	0.006	1.116	1.032-1.206
E_2_	-0.085	0.020	17.411	0.000	0.918	0.882-0.956
P/E_2_	0.115	0.032	8.515	0.003	1.254	1.074-1.586

**Fig.1:**
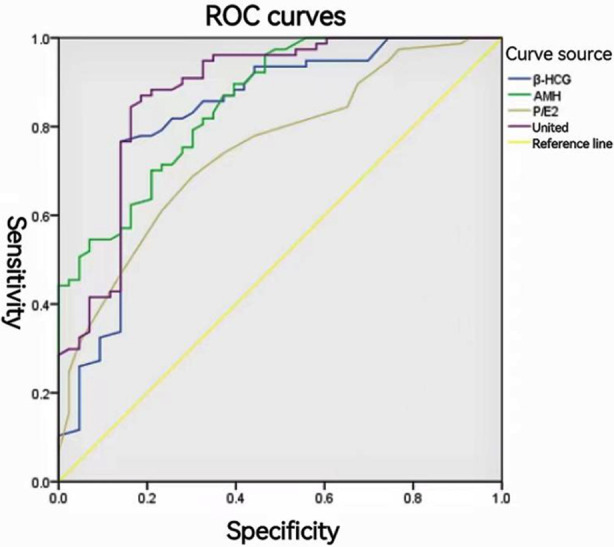
ROC of serum β-HCG, AMH, P/E_2_ for predicting pregnancy outcome.

Plotting the ROC curves revealed an AUC of 0.83 (95% CI:0.741-0.910) for serum β-HCG, 0.86 (95% CI:0.788-0.922) for AMH, 0.75 (95% CI:0.662-0.838) for P/E_2_, and the combination of these three to predict thin uterine The AUC for freeze-thaw embryo transfer FET cycle pregnancy rate in patients with endometrium was 0.88 (95% CI:0.807-0.947). The results are shown in Table-V and Fig.1.

**Table-V:** The value of serum β-HCG, AMH and P/E_2_ in predicting pregnancy outcome.

Factors	AUC	cut-off	95% CI	P	Specificity	Sensitivity	Jorden Index
β-HCG	0.826	5.152U/L	0.741-0.910	0.000	0.818	0.723	0.541
AMH	0.855	2.435ng/ml	0.788-0.922	0.000	0.838	0.715	0.553
P/E_2_	0.750	0.267	0.662-0.838	0.000	0.725	0.632	0.357
United	0.877	-	0.807-0.947	0.000	0.873	0.718	0.591

## DISCUSSION

The pathophysiological features of thin endometrium mainly lie in high resistance to uterine arterial blood flow, slow growth of glandular epithelium, vascular dysplasia and low expression of vascular endothelial growth factor. Its pathogenesis is mostly related to endometrial injury and endocrine disorders.^10^ It was found that thin endometrium is closely related to pregnancy outcome of embryo implantation.^11^ The results of this study showed that only 77 (64.17%) of 120 patients with thin endometrium had a successful pregnancy during the FET cycle, while 43 (35.83%) did not. Zhang J^12^ et al. included a total of 6181 patients and grouped them according to endometrial thickness, and found that compared to the group with endometrial thickness greater than or equal to 10 mm, the group with endometrial thickness less than 8 mm had FET cycle pregnancy rate, implantation rate, neonatal birth weight decreased and miscarriage rate increased. Further multiple linear regression analysis revealed that endometrial thickness less than 8 mm was an independent predictor of neonatal birth weight. Shaodi Z^13^ et al. similarly found that a thin endometrium reduced pregnancy and live birth rates in FET cycles. This may be due to the low expression of vascular endothelial growth factor and high blood flow resistance in thin endometrium during the formation of uterine embryo circulation, which is not conducive to spiral arteriole recast, reducing placental blood volume and restricting fetal growth. In addition, in patients with the absence or thin endometrial functional layer, the embryo implantation is very close to the basal layer and spiral artery, which increases the oxygen content of the endometrial surface, while the high oxygen tension of the endometrial is not conducive to the formation of the placenta and embryo implantation.^14^ Therefore, exploring a specific diagnostic target or group of targets is key to improving the reproductive prognosis of ART in patients with thin endometrium.

β-HCG is a glycoprotein hormone secreted by placental syncytiotrophoblast cells, which has physiological effects such as maintaining the luteal function in early pregnancy. In this study, β-HCG and AMH were higher in the pregnancy group than in the non-pregnancy group, and the best cut-off values were taken as 5.152 U/L and 2.435 ng/ml, respectively, with the highest predictive value. A retrospective cohort study by Wu M et al.^15^ showed that β-hCG levels on day 12 after FET were significantly associated with clinical pregnancy. The critical values of β-hCG in blastocyst and blastocyst groups were 156.60 mIU/mL and 217.70 mIU/mL, respectively. This conclusion supports the results of our study, but the optimal threshold for β-HCG is higher than that of our study. It may be because blood was drawn at the date of FET transplantation in this study, whereas blood was drawn 12 days after FET transplantation in their study. In addition, Kamel HM et al.^16^ found that serum AMH had a good predictive value for embryo quality and pregnancy rates in infertile patients undergoing intracytoplasmic sperm injection (ICSI) cycles. Serum AMH levels at a threshold of 2.8 μg/L are more sensitive and specific, and can predict the occurrence of good embryo quality and chemical or clinical pregnancy. Which supports the conclusion of this study. The reason for these results may be that AMH may affect endometrial preparation by regulating collagen and fibrotic processes, or endometrial receptivity by altering uterine artery flow. In addition, AMH can prevent the initial recruitment process of primal follicles, avoid premature depletion of follicle pool, promote follicle growth and development, improve ovarian function and fertility of women of reproductive age, and enhance the success rate of FET pregnancy. Under the stimulation of β-HCG, the luteum of pregnancy secretes a large amount of progesterone, promotes the proliferation of trophoblast cells, and is conducive to embryonic development.

During embryo implantation, the uterus and blastocyst secrete relevant proteins and factors in a strict temporal and spatial sequence in a highly coordinated manner, assisting embryo implantation through interaction.^17^ The AUC of P/E_2_ for predicting the pregnancy rate of FET cycles in patients with thin endometrium was 0.750 (95% CI:0.662-0.838), with the highest predictive value when the optimal cut-off value was 0.267, and the specificity and sensitivity were 0.725 and 0.632, respectively, suggesting that P/E_2_ is a potential biological indicator for predicting the success rate of FET pregnancies. Patients with persistently or significantly lower P/E_2_ values may be at high risk of pregnancy failure and should be put on high alert.

### The reasons for this may be:

1. E_2_ is an estrogen that is secreted by the ovarian follicles and regulates the development of secondary sexual characteristics and female reproductive organs. low levels of E_2_ can reduce endometrial tolerance and delay follicular development. Lee M^18^ et al. reported that estrogen plays an important role in the synthesis of progesterone receptors and low levels of E_2_ can affect the amount of progesterone receptor synthesis and endometrial tolerance in the implantation window, causing failure of implantation.
2. P is a progestin that is produced and secreted by follicular cells and luteal granulosa cells. Its binding to specific receptors can lead to interstitial metaplasia, which induces a secretory response from endometrial glands, relaxes uterine fibres and inhibits uterine contraction and excitability, thereby establishing stable maternal-fetal immune tolerance.^19^ Massimiani M^20^ et al. reported that cytosolic synapses are ultrastructural markers of endometr
3. High levels of oestrogen can impair the function of the corpus luteum, causing the endometrium to mature out of sync with embryonic development and weakening the response to progesterone. Low oestrogen may prolong the opening of the endometrial implantation window, so maintaining a stable P/E_2_ is important for improving endometrial tolerance, defining the “implantation window” and increasing the success rate of pregnancy.
4. There have been more studies on the correlation between MAH or HCG and pregnancy outcomes for different causes of infertility, but fewer studies on their role in FET or thin endometrium. This study innovatively fills the gap in related research fields and provides reliable clinical data for subsequent studies.

### Limitations:

It includes a small number of patients. In view of this, more patients should be included in future studies to further validate the findings of this study.

## CONCLUSIONS

In conclusion, the FET pregnancy rate in patients with thin endometrium is at an intermediate level. Serum β-HCG, AMH and P/E_2_ are closely related to the pregnancy rate in FET cycles in patients with thin endometrium, so changes in the three indicators should be monitored dynamically in clinical practice.

### Recommendations:

In addition, further studies with large samples and multiple centers are needed to explore the most suitable endometrial preparation scheme for FET patients with thin endometrial.

### Authors’ Contributions:

**YY:** Carried out the studies, participated in collecting data, and drafted the manuscript, and are responsible and accountable for the accuracy or integrity of the work.

**JZ, WL** and **XY:** Performed the statistical analysis and participated in its design. Critical Review.

All authors have read and approved the final manuscript.
